# Rapid Diagnosis of Pulmonary and Extrapulmonary Tuberculosis in HIV-Infected Patients. Comparison of LED Fluorescent Microscopy and the GeneXpert MTB/RIF Assay in a District Hospital in India

**DOI:** 10.1155/2012/932862

**Published:** 2012-08-26

**Authors:** Gerardo Alvarez-Uria, Jose M. Azcona, Manoranjan Midde, Praveen K. Naik, Srinivasulu Reddy, Raghuprakash Reddy

**Affiliations:** ^1^Department of Infectious Diseases, Rural Development Trust Hospital, Kadiri Road, Anantapur District, Andhra Pradesh, Bathalapalli 515661, India; ^2^Department of Microbiology, Rural Development Trust Hospital, Kadiri Road, Anantapur District, Andhra Pradesh, Bathalapalli 515661, India

## Abstract

HIV-related tuberculosis is difficult to diagnose and is associated with high morbidity and mortality. Recently, the World Health Organization has endorsed the GeneXpert MTB/RIF (Xpert) assay for the diagnosis of pulmonary tuberculosis in HIV-infected patients from developing countries, but information about the use of Xpert for the diagnosis of extrapulmonary tuberculosis is scarce. In this study, we compared the performance of light-emitting diode (LED) auramine fluorescent microscopy and the Xpert assay for the diagnosis of tuberculosis in HIV infected patients in a district hospital of India. Although at higher cost, Xpert outperformed LED fluorescent microscopy in all type of specimens, especially in cerebrospinal fluid where the number of positive results was increased 11 times. Pleural fluid, ascitic fluid, pus, and stool specimens also yielded positive results with the Xpert assay. When collecting two additional early-morning sputum samples, the increase of the number of positive results with the Xpert assay was lower than previously reported for HIV infected patients. Rifampicin resistance was observed in 2.2% of the cases. The results of this study show that the Xpert assay can dramatically improve the rapid diagnosis of tuberculous meningitis and other types of extrapulmonary tuberculosis of HIV infected patients.

## 1. Introduction

In 2010, there were 350,000 tuberculosis-related deaths in HIV-infected people, most of them in developing countries [1]. One of the most important reasons for this high number of deaths is the difficulty of diagnosing tuberculosis in the HIV population [2, 3]. There is an urgent need for implementing new diagnostic methods for tuberculosis in resource-limited setting with high HIV prevalence. 

Microbiological identification of *Mycobacterium tuberculosis* from cultures is the gold standard for diagnosing tuberculosis infection. However, culture of mycobacteria is not able to provide a rapid diagnosis for the clinical management of severe cases and requires expensive and sophisticated laboratory facilities that cannot be afforded in most of resource-limited settings.

The World Health Organization (WHO) has recently endorsed the implementation of light-emitting diode (LED) fluorescent microscopy and the GeneXpert MTB/RIF assay for national tuberculosis programmes in developing countries [4, 5]. LED fluorescent microscopy is less expensive than the conventional fluorescence microscopy, has been shown 84% sensitivity (95% confidence interval [CI], 76 to 89) and 98% specificity (95% CI, 85 to 97) against culture as the reference standard, and has been shown to improve by 6% (95% CI, 0.1 to 13%) the sensitivity compared to the traditional Ziehl-Neelsen microscopy [5, 6]. The Xpert MTB/RIF is a new fully automated diagnostic molecular test with an analytic sensitivity of five genome copies of purified DNA and 131 cfu/ml of *M. tuberculosis* in sputum and, moreover, is able to detect more than 99.5% rifampicin resistance mutations, an indicator of multidrug-resistant tuberculosis, in less than two hours [7]. The Xpert MTB/RIF assay requires minimal biosafety infrastructure and training, and data from controlled clinical validation studies has shown a sensitivity of 92% compared to culture utilizing a single specimen [7]. However, these data come from clinical trials, and information about the performance of LED fluorescent microscopy and Xpert MTB/RIF in real-life situations is desirable before worldwide implementation. 

Although pulmonary tuberculosis is more common than extrapulmonary tuberculosis, extrapulmonary tuberculosis can be present in up to 40% of HIV-infected patients with tuberculosis [8]. In this study, we show the experience of utilizing LED fluorescent microscopy and Xpert MTB/RIF in a district hospital from rural India for the diagnosis of pulmonary and extrapulmonary tuberculosis in a cohort of HIV-infected patients. 

## 2. Methods

The study was performed in Bathalapalli RDT Hospital, a district hospital in the rural district of Anantapur, Andhra Pradesh, India. From May 2011 to January 2012, HIV-infected patients with suspicion of tuberculosis were admitted in the Department of Infectious Diseases [9]. 

According to the suspected localization of the tuberculosis infection, body specimens from these patients were sent to the Microbiology Department. There, the same specimen was processed for LED fluorescent microscopy and for Xpert MTB/RIF test. Additionally, if pulmonary tuberculosis was suspected, two additional sputum samples collected in the early morning were sent for LED fluorescent microscopy following current WHO recommendations [10]. Smears for LED fluorescent microscopy were prepared following standard procedures of preparation of slides and staining with auramine [11]. Extrapulmonary specimens were concentrated by cytocentrifugation, but we did not use any concentration method for sputum. All smears reported as “scanty” acid-fast bacilli (AFB) by LED fluorescent microscopy were considered as positive results for performing the analysis of the study. Sample preparation and Xpert MTB/RIF procedure were performed by trained operators as described previously [12, 13]. 

Statistical analysis was performed using Stata Statistical Software (Stata Corporation. Release 11. College Station, TX, USA). Confidence intervals for the absolute difference and ratios between the two tests were calculated using the McNemar's test. The study was approved by the Ethical Committee of the RDT Institutional Review Board. 

## 3. Results 

During the period of the study, 518 samples from patients with suspicion of tuberculosis were sent to the Microbiology Department for performing Xpert MTB/RIF. Of all sputum specimens, 79 were rejected as they were considered to be saliva. Of the 439 processed specimens, 12 (2.7%) were not able to yield a valid result. The proportion of invalid results were 3/12 (25%) for stool, 1/11 (9.1%) for pus, 1/19 (5.3%) for ascitic fluid, 3/148 (2%) for cerebrospinal fluid, 3/169 (1.8%) for sputum, and 1/80 (1.3%) for pleural fluid. Of 427 specimens that yielded a valid Xpert MTB/RIF result, 3 (0.7%) did not yield a valid result for rifampicin resistance. LED fluorescent microscopy was not performed in eight specimens. Of the initial 518 specimens, 419 (81%) were included in the analysis.

The median age of patients was 35.2 (interquartile range, 30 to 41.2) years, 121 (28.9%) were women and the median CD4 lymphocyte count was 128 (interquartile range, 59 to 266). The number of positive results by type of sample is presented in Table 1. The majority of specimens came from sputum, CSF, and pleural fluid of the patients. Globally, the use of Xpert MTB/RIF assay increased the number of positive results by 16.5%, but this increase was more important for extrapulmonary than for pulmonary specimens. The highest increase in positive results was seen in CSF followed by ascitic fluid and pleural fluid. Although we found also a significant increase in positive results comparing Xpert MTB/RIF with LED fluorescent microscopy when performing both test in the same sputum specimen, when collecting two sputum specimens for LED fluorescent microscopy, the increase of positive results was more modest. Nine (2.16%) out of 416 specimens with valid rifampicin test were found to be rifampicin resistant, six from sputum, one from pus, one from CSF, and one from pleural fluid. All 26 specimens that were reported as “scanty AFB” in the sputum smear yielded a positive result in the Xpert MTB/RIF assay.

Data about the costs, consumption of time for processing one sample in our laboratory, rifampicin resistance information, maximum number of samples processed per hour, and requirement of external quality control, and operator training of the two assays are given in Table 2. The cost of the equipment, annual maintenance, and processing one sample is 8, 3, and 82 times higher in the Xpert assay, respectively. Although the Xpert assay does not require operator expertise or external quality controls and is able to provide information about rifampicin resistance, only four samples can be processed every two hours.

## 4. Discussion 

Although Xpert MTB/RIF was initially validated only for pulmonary specimens, the result of this study shows that the Xpert MTB/RIF assay can increase more than three times the rapid diagnosis of extrapulmonary tuberculosis compared to LED fluorescent microscopy. Extrapulmonary tuberculosis is more common in HIV-infected patients than in the general population regardless of the CD4 lymphocyte count and has been associated with high morbidity and mortality [14]. Diagnosis of extrapulmonary tuberculosis in HIV and non-HIV-infected patients is challenging because of the lack of rapid diagnostic tools, especially in limited-resource settings where the traditional Ziehl-Neelsen microscopy is frequently the only method available [15–17]. Delay in the initiation of therapy is strongly associated with death and sequelae, particularly in tuberculous meningitis [18–20]. This is especially important as in this study, the highest increase in the number of positive results was seen in CSF specimens. Implementation of the Xpert MTB/RIF assay in developed and developing countries can improve the clinical management of HIV-infected individuals with suspicion of extrapulmonary tuberculosis.

We found that all sputum smears reported as “scanty AFB” had a positive Xpert result. Other studies have also found very low rate of false positive results in settings with high prevalence of tuberculosis in HIV and non HIV-infected patients with “scanty AFB” in sputum smears [21, 22]. Knowing the high morbidity and mortality of HIV-related tuberculosis, these patients should be considered as infected by tuberculosis until proven otherwise.

The Xpert MTB/RIF assay outperformed LED microscopy in all types of specimens. However, the cost the equipment, the annual maintenance, and the consumables are considerably higher in the Xpert MTB/RIF assay. One important limitation of the Xpert assay is that it can process a maximum of four samples every two hours, so it may not be suitable for busy laboratories receiving large number of samples in resource-limited setting. 

The study has some limitations. We did not perform culture for mycobacteria to rule out the possibility of false positive results. However, both LED fluorescent microscopy and Xpert MTB/RIF have shown specificities above 95% in previous studies [7, 13, 17, 23], so the possibility of bias due to false positive results is very small. In the present study, the results of Xpert MTB/RIF with sputum specimens were more modest than previously reported. In a previous study performed in South Africa with HIV-infected patients, the Xpert MTB/RIF increased case detection for tuberculosis by 45% compared to fluorescent microscopy [24]. The difference between both studies may be explained by the difference in the populations of the studies and in the way the sputum specimens were collected. In the South-African study, two sputum samples were collected in a single visit to outpatient clinics before the initiation of antiretroviral treatment and regardless of symptoms [24]. In our study, we studied patients with high suspicion of active tuberculosis infection, and all patients were admitted to the hospital. WHO strongly recommend the use of Xpert MTB/RIF for the diagnosis of HIV associated pulmonary tuberculosis [4]. However, the cost of Xpert MTB/RIF can be too high for some resource-limited settings. According to the results of this study, in settings with low prevalence of multidrug-resistant tuberculosis where it is possible to obtain two additional early-morning sputum samples in different days, LED fluorescent microscopy could be used with not much loss of sensitivity compared to the Xpert MTB/RIF assay for the diagnosis of HIV-infected patients with suspicion of pulmonary tuberculosis. For example, due to economical constraints and an increasing number of samples coming to our laboratory, we decided to restrict the use of the Xpert MTB/RIF assay for the diagnosis of extrapulmonary tuberculosis and for investigating rifampicin resistance in smear-positive patients with risk factors for having drug-resistant tuberculosis (previous antituberculosis treatment or contact with patients with drug resistant tuberculosis).

## 5. Conclusions

The results of this study indicate that the implementation of the Xpert MTB/RIF assay could dramatically improve the rapid diagnosis of extrapulmonary tuberculosis in HIV-infected patients, especially in cases with suspicion of tuberculous meningitis. In settings where Xpert cannot be afforded, LED fluorescent microscopy may be used for the diagnosis of pulmonary tuberculosis with acceptable results compared to the Xpert MTB/RIF assay if several sputum specimens can be collected in different days.

## Figures and Tables

**Table 1:** Positive results utilizing light-emitting diode fluorescent microscopy and Xpert MTB/RIF assay.

	Total	Smear positive		Xpert positive		Absolute difference	Ratio Xpert/LED
	*N*	*N*	% (95% CI)	*N*	% (95% CI)	% (95% CI)	(95% CI)
Sputum	166	106	63.9 (56.2 to 70.8)	124	74.7 (67.5 to 80.8)	10.8 (5.2 to 16.5)	1.17 (1.08 to 1.26)
Sputum x3	166	116	69.9 (62.4–76.4)	124	74.7 (67.5 to 80.8)	4.8 (0.6 to 9.1)	1.07 (1.02 to 1.13)
Extrapulmonary	253	23	9.1 (6.1 to 13.3)	74	29.2 (24 to 35.2)	20.2 (14.8 to 25.5)	3.22 (2.29 to 4.52)
CSF	142	3	2.1 (0.7 to 6.4)	35	24.6 (18.2 to 32.4)	22.5 (15 to 30.1)	11.67 (3.95 to 34.42)
Pleural fluid	75	10	13.3 (7.3 to 23.1)	24	32 (22.4 to 43.4)	18.7 (8.5 to 28.8)	2.4 (1.49 to 3.85)
Ascitic fluid	18	2	11.1 (2.8 to 35.4)	5	27.8 (12 to 52)	16.7 (−6.1 to 39.4)	2.5 (0.85 to 7.31)
Pus	10	7	70 (37.5 to 90.1)	8	80 (45.8 to 95)	10 (−18.6 to 38.6)	1.14 (0.88 to 1.49)
Stool	8	1	12.5 (1.7 to 53.9)	2	25 (6.3 to 62.4)	12.5 (−22.9 to 47.9)	2 (0.5 to 8)
Total	419	129	30.8 (26.5 to 35.4)	198	47.3 (42.5 to 52.1)	16.5 (12.6 to 20.3)	1.53 (1.38 to 1.7)

CI: confidence interval; LED: light-emitting diode fluorescent microscopy; CSF: cerebrospinal fluid.

**Table 2:** Comparison of costs, features, and requirements of the two assays

	Xpert MTB/RIF	LED microscopy
Cost of equipment (USD)*	17790^†^	2136
Cost per Sample (USD)	16.84*	0.2^‡^
Annual maintenance (USD)	1600*	500
Average time consumed per sample (minutes)	4	4^§^
Rifampicin resistance information	Yes	No
Maximum number of samples processed per hour	2	30
External quality control required	No	Yes
Operator expertise required	No	Yes

LED: light-emitting diode; *negotiated prices for developing countries, prices extracted from http://www.finddiagnostics.org/about/what_we_do

/successes/find-negotiated-prices/ (accessed on July 19, 2012) but transport and shipping (international and local costs), insurance, clearing, customs duties, and local taxes, where applicable, as well as the local representative costs are not included; ^†^includes uninterrupted power supply; ^‡^Himedia fluorescent stain kit for mycobacteria; ^§^based on staining 30 samples at one time.
